# Association of interarm blood pressure difference with selected body circumferences among Walter Sisulu University community

**DOI:** 10.1186/s12889-024-18117-5

**Published:** 2024-02-29

**Authors:** Wenzile S. Mthethwa, Nthai E. Ramoshaba, Zuqaqambe M. Mampofu

**Affiliations:** Department of Human Biology, Walter Sisulu University, Nelson Mandela Drive, Mthatha, 5117 South Africa

**Keywords:** Interarm blood pressure difference, Anthropometry, Body circumferences, Vascular diseases, Walter Sisulu University community

## Abstract

**Background:**

A high interarm blood pressure difference (IAD) has been identified as a precursor of vascular diseases. Anthropometric measures for obesity such as body circumferences including waist circumference (WC), mid-upper arm circumference (MUAC) and neck circumference (NC) have been associated with a high IAD in Western countries. However, the prevalence of IAD and its association with body circumferences in South African communities such as universities is not well established. Therefore, this study aimed at investigating the correlation of IAD with selected body circumferences among the Walter Sisulu University (WSU) community.

**Methods:**

A total of 230 participants, 117 males and 113 females, consisting of 185 students and 45 staff members from WSU, aged 18–27 and 18–63 years respectively, participated in this cross-sectional study. The selected body circumferences: WC, MUAC, and NC were measured using standard procedures. Systolic blood pressure (SBP) and diastolic blood pressure (DBP) were measured in both arms simultaneously using automated machines. IAD was achieved by calculating differences in SBP and DBP between the left (L) and right (R) arms, (R -L), and getting the absolute value, L–R (|L–R|).

**Results:**

14.78% of the participants had an interarm SBP difference (IASBPD) ≥ 10 mmHg, and 4.35% of participants had an interarm DBP difference (IADBPD) ≥ 10 mmHg.

In a Pearson’s correlation analysis, IASBPD was positively correlated with the selected body circumferences (WC, *r* = 0.29; *P* < 0.001; MUAC, *r* = 0.35; *P* < 0.001; NC, *r* = 0.27; *P* < 0.001) and mean arterial pressure (MAP) (*r* = 0.30; *P* < 0.001). In the multivariable-adjusted regression analyses, IASBPD was positively associated with MUAC (adjusted *R*^2^ = 0.128, β = 0.271 (95% CI = 0.09; 0.60), *P* = 0.008), and NC (adjusted *R*^2^ = 0.119, β = 0.190 (95% CI = 0.01; 0.32), *P* = 0.032) only, adjusted for MAP, age, gender, body mass index, smoking, and alcohol. There was no association of body circumferences with IADBPD.

**Conclusion:**

A high IAD is common among students and staff members of the WSU community. Furthermore, IAD showed a positive correlation with MUAC and NC. These body circumferences can serve as indicators of high IAD, aiding in the early detection and prevention of vascular diseases.

**Supplementary Information:**

The online version contains supplementary material available at 10.1186/s12889-024-18117-5.

## Introduction

Blood pressure (BP) measurements play a crucial role in the evaluation and management of hypertension, a major contributor to cardiovascular diseases (CVDs) development. Guidelines for the management of hypertension recommend measuring BP in both arms, as large differences between arms may exist, [1–3].

The difference in blood pressure between the arms is known as interarm blood pressure difference (IAD) and this term dates centuries ago by [4], and can arise from both physiological and pathological factors. In young adults, IAD may occur due to muscle compression of the artery supplying the arm or an obstruction within the artery that causes turbulent blood flow [5]. In older people, it is usually due to blood blockage caused by atherosclerosis [6] and is characterized by a systolic interarm blood pressure difference (IASBPD) greater than 10 mmHg [7, 8].

IAD has caught more attention in recent years due to researchers suggesting that high IAD is predictor of numerous vascular diseases, including but not limited to peripheral vascular diseases, cerebrovascular diseases, and connective tissue disorders [9]. In addition, it has been found that higher IAD is associated with increased risk of cardiovascular incidents and obesity- a major atherosclerosis risk [10, 11], and can give rise to increased morbidity, and mortality rates in individuals [7, 8]. It is therefore, of paramount importance to identify modifiable risk factors to curb cardiovascular morbidity and mortality.

Most studies have relied on body mass index (BMI) as the measure of obesity [12, 13]. However, BMI does not distinguish between lean mass and fat mass and can overestimate the degree of obesity in individuals who are extremely muscular [14]. Additionally, BMI does not give information about fat distribution, which is crucial in determining the risk for atherosclerosis [15, 16]. Given the circumstances of the vascular disease risks, this study explored other anthropometric variables to assess adiposity which included waist circumference (WC), mid-upper arm circumference (MUAC) and neck circumference (NC), body circumferences as for atherosclerotic cardiovascular diseases in individuals as compared with BMI [17–19].

Few studies have reported a positive association between NC, MUAC, and WC with IAD in middle-aged individuals from Western countries, [10, 20] . However, no studies have been conducted to investigate the proportion of IAD and its relationship with body circumferences in the general population of South Africa, in the Eastern Cape Province. It is crucial to establish the proportion of IAD and its relationship with body circumferences among WSU community as this can help identify individuals who are at high risk and improve early detection and prevention of vascular diseases. The present study investigated the association between IAD and selected body circumferences among Walter Sisulu University (WSU) community.

## Methods

### Study design and data collection

This cross-sectional study was conducted in one of South Africa’s historically disadvantaged universities, WSU, located in Mthatha in the Eastern Cape Province. WSU includes approximately 97% Black, 2% Indian, and 1% White individuals in terms of ethnic composition. A total of 230 participants (males, *n* = 117; females, *n* = 113) from students (*n* = 185, age = 18–27 years), 45 staff members (*n* = 45, age = 18–63 years), participated in this cross-sectional study. A power calculation revealed that a minimum sample size of *n* = 89 would be required to perform our multivariable regression analysis with an effect size of 0.15, alpha set to 0.05, and power to 0.95.

#### Inclusion/exclusion criteria

Students and staff members both males and females from WSU aged 18 years and above with no history of cardiovascular, renal, and endocrine diseases were recruited to participate in this study. Furthermore, pregnant women were excluded from the study.

#### Recruitment

Participants were recruited through word-of-mouth from their respective residencies and offices to the physiology laboratory where the data were collected.

#### Data collection

Each participant completed a general demographic and lifestyle questionnaire, regarding age, gender, ethnicity, self-reported smoking, and self-reported alcohol consumption. One of the authors, N.E. Ramoshaba conducted the face validity for the questionnaires. The language and content were deemed appropriate (simple and easy to understand) and relevant to our study.

### Measurements

#### Anthropometric measurements

Anthropometric measurements were carried out by researchers and well-trained assistants in accordance with the guidelines of the International Society for the Advancement of Kinanthropometry [21]. The neck, waist, and mid-upper arm circumferences were measured to the nearest 0.1 cm using a flexible steel tape (Lufkin Steel Tape; W606PM; Lufkin, TX, USA; Apex, NC, USA). The participants were required to stand during the measurement with their heads kept in the Frankfort plane. The NC was measured perpendicular to the long axis of the neck, directly above the thyroid cartilage. Since the tissues in this area are compressible, pulling the tape too tight when measuring was strictly avoided. WC measurements were taken at the level of the narrowest point between the iliac crest and the bottom part of the thoracic cage, with the participants’ arms folded across the thorax and standing in an upright relaxed position after mild expiration.

MUAC measurements were taken while the participants were settled in a comfortable position with their arms at their sides. The tape measure was positioned perpendicular to the long axis of the humerus, where the mid acromiale-radiale was marked. MUAC measurements were then taken at that area, while the muscles of the arm were relaxed.

A SECA 213 Portable Stadiometer was used to measure the body height to the nearest 0.1 cm (SECA, Hamburg, Germany). The participants stood with their feet together and their heels, buttocks, and upper back touching the scale for body height measurements. The participants were instructed to take a deep breath and hold it while keeping their head in the Frankfort plane. A gentle upward lift through the mastoid processes was applied. The stadiometer's base was then lowered to the vertex of the head, and if there was a lot of hair on the head, a small amount of pressure was applied to touch the top of the head.

Using an electronic scale, the body weight was measured to the nearest 0.1 kg (SECA, Hamburg, Germany). The scale reading was checked before the participants could climb onto it, the participants then stood on the center of the scale without support and with their weight evenly distributed on both feet. Their heads were tilted upwards, and their eyes fixed forward. The readings were then taken. Body mass index (weight (kg)/height (m)2) was calculated.

#### Blood pressure measurements

Omron M3 BP monitors were used to assess clinic blood pressure on both arms, simultaneously (Omron, Kyoto, Japan). After the participants had been seated for at least 5 min, three readings of systolic blood pressure (SBP), diastolic blood pressure (DBP), and heart rate were taken on both arms simultaneously. All three readings for each arm were recorded. Mean arterial pressure (MAP) was calculated [SBP + (2 × DBP)]/3. Differences in BP were calculated by subtracting the left-arm BP (L) from the right-arm BP (R). Absolute BP difference or absolute value of R –L (|R –L |) was calculated to determine the difference between left-arm BP and right-arm BP regardless of which arm showed a higher BP value [10]. A large inter-arm BP difference was defined as an absolute interarm BP difference of greater than 10 mmHg [22, 23]. The BP was categorized into prehypertension (SBP = 130–139, DBP = 80–89 mmHg),and hypertension (SBP ≥ 140 mmHg or DBP ≥ 90 mmHg) depending on the arm with the higher values [3].

### Statistical analysis

The formal test (Kolmogorov–Smirnov test) and graphical approaches were used to analyze normal data distribution. Continuous data was presented as mean ± standard deviation after assessing for normal distribution. Independent t-test analysis was used to test the differences in continuous variables according to IAD status. Categorical data was analyzed according to IAD status using chi-square test and presented as percentages. Pearson’s correlation analysis was used to determine the relationship between IAD, NC, WC, and MUAC. Multiple regression analyses were used to investigate the independent association of IAD as dependent variable with body circumferences as independent variables, covariates for all multiple regression models were age, gender, MAP, BMI, alcohol, and smoking. All the statistical analyses were performed using the Statistical Package for the Social Sciences (SPSS Inc., Chicago, IL, USA, 26.0). The statistical significance was set at *P* < 0.05.

## Results

Table S1 shows that 14.78% of the participants had IASBPD ≥ 10 mmHg, and 4.35% of participants had an IADBPD ≥ 10 mmHg. Splitting by gender, 14.5% males and 15% females had IASBPD ≥ 10 while 6% males and 2.7% females had IADBPD ≥ 10. Table 1 shows the characteristics of the WSU community by IAD status. IAD ≥ 10 mmHg for both SBP and DBP was present in older participants (IASBPD, 29 vs. 23.39 years *P* < 0.001; IADBPD, 30.20 vs. 23.95 years) as compared to younger participants. Participants with IASBPD ≥ 10 mmHg showed higher body circumferences (WC; 85.00 vs 76.59 cm *P* < 0.001; MUAC, 31.92 vs 28.36 cm *P* < 0.001; NC, 37.37 vs 33.99 cm *P* < 0.001), BMI (28.29 vs 24.88 kg/m^2^), MAP (99.1 vs 92.63 mmHg), and proportion of prehypertension and hypertension (26.5% vs 18.4 and 41.2 vs 13.3% *P* < 0.001 respectively) than participants with IASBPD ≤ 10 mmHg.

**Table 1:** Baseline characteristics by systolic and diastolic interarm blood pressure difference of WSU community

	Total (mmHg)	SBP (right-left)			DBP (right-left)		
	(*n* = 230)	≤ 10 mmHg (*n* = 196)	≥ 10 mmHg (*n* = 34)	*p*-value	≤ 10 mmHg (*n* = 220)	≥ 10 mmHg (*n* = 10)	*p*-value
Characteristics							
Gender (Male %)	117 (50.9)	100 (85.5)	17 (14.5)	0.913	110 (94.0)	7 (6.0)	0.216
Age (years)	24.22 ± 8.44	23.39 ± 6.81	29 ± 13.90	< 0.001	23.95 ± 8.12	30.20 ± 12.99	0.022
WC (cm)	77.81 ± 12.33	76.59 ± 11.26	85.00 ± 15.60	< 0.001	77.54 ± 11.98	83.77 ± 18.20	0.118
MUAC (cm)	28.89 ± 4.47	28.36 ± 3.98	31.92 ± 5.80	< 0.001	28.86 ± 4.45	29.61 ± 5.00	0.603
NC (cm)	34.51 ± 5.22	33.99 ± 3.99	37.37 ± 9.19	< 0.001	34.39 ± 5.21	36.94 ± 5.09	0.132
BMI (kg/m^2^)	25.39 ± 5.93	24.88 ± 5.49	28.29 ± 7.47	0.002	25.34 ± 5.89	26.26 ± 6.91	0.634
MAP (mmHg)	93.60 ± 11.81	92.63 ± 11.18	99.16 ± 13.84	0.003	93.42 ± 11.71	97.51 ± 13.86	0.285
**Hypertension**							
Prehypertensive, n (%)	145 (63.0)	36 (18.4)	9 (26.5)	< 0.001	42 (19.1)	3 (30)	0.468
Hypertensive, n (%)	85 (37)	26 (13.3)	14 (41.2)		37 (16.8)	3 (30)	
**Lifestyle**							
Smoking, n (%)	56 (24.3)	49 (25.00)	7 (20.6)	0.580	52 (23.6)	4 (40)	0.238
Alcohol, n (%)	125 (54.3)	109 (55.6)	16 (47.1)	0.355	121 (55)	4 (40)	0.352

*Abbreviations*: *SBP* Systolic blood pressure, *DBP* Diastolic blood pressure, *WC* Waist circumference, *MUAC* Mid upper arm circumference, *NC* Neck circumference, *BMI* Body mass index, *MAP* Mean arterial pressur

Furthermore, in the Pearson correlation analysis (Table 2), IASBPD showed a significant positive correlation with the selected body circumferences (WC, *r* = 0.29; *P* < 0.001; MUAC, *r* = 0.35; *P* < 0.001; NC, *r* = 0.27; *P* < 0.001) and MAP (*r* = 0.30; *P* < 0.001). NC showed a significant correlation with IADBPD (*r* = 0.14; *P* = 0.035). Table 3 shows the multivariable-adjusted regression analysis models’ results for body circumferences, MUAC and NC remained positively associated with IASBPD (MUAC, adjusted *R*^2^ = 0.128, β = 0.271 (95% CI = 0.09; 0.60), *P* = 0.008), (NC, adjusted *R*^2^ = 0.119, β = 0.153 (95% CI = 0.01; 0.32), *P* = 0.032), WC did not show any association with IASBPD adjusted for age, gender, MAP, BMI, smoking, and alcohol. Table S2 shows multivariable-adjusted regression analysis models’ results for body circumferences, all the selected body circumferences showed no significant association with IADBPD. Table S3 presents multivariable-adjusted regression analyses results including all the selected body circumferences, only MUAC showed a positive association with IASBPD (adjusted *R*^2^ = 0.130, β = 0.2421 (95% CI = 0.03; 0.60), *P* = 0.031) after adjusting for WC, NC, MAP, age, gender, BMI, smoking, and alcohol.

**Table 2:** Pearson correlation between interarm blood pressure diffrence and body circumferences

Variables	Interarm blood pressure difference	
	SBP (right-left)	DBP (right-left)
WC (cm)	*r* = 0.29; *P* < 0.001	*r* = 0.08; *P* = 0.246
MUAC (cm)	*r* = 0.35; *P* < 0.001	*r* = 0.06; *P* = 0.356
NC (cm)	*r* = 0.27; *P* < 0.001	*r* = 0.14; *P* = 0.035
MAP (mmHg)	*r* = 0.30; *P* < 0.001	*r* = 0.12; *P* = 0.067

*Abbreviation*: *WC* Waist circumference, *MUAC* Mid upper arm circumference, *NC* Neck circumference, *MAP* Mean arterial pressure

**Table 3:** Independent association between interarm systolic blood pressure difference as dependent variable and WC, MUAC and WC as independent variables

	Interarm systolic blood pressure difference							
	Adjusted *R*^2^ = 0.101		Adjusted *R*^2^ = 0.128			Adjusted *R*^2^ = 0.119		
	β (95% CI)	*P*-value		β (95% CI)	*P*-value		β (95% CI)	*P*-value
WC (cm)	0.055 (-0.09;0.15)	0.672	MUAC (cm)	0.271 (0.09;0.60)	0.008	NC (cm)	0.153 (0.01;0.32)	0.032
MAP (mmHg)	0.186 (0.02;0.16)	0.013		0.170 (0.01;0.15)	0.021		0.147 (0.05;0.14)	0.053
Age (years)	0.069 (-0.06;0.15)	0.385		0.050 (-0.07;0.13)	0.502		0.070 (-0.50;0.15)	0.349
Gender (male & female)	0.027 (-1.88;1.26)	0.700		-0.009 (-1.63;1.42)	0.890		0.007 (-1.50;1.66)	0.921
BMI (kg/m^2^)	0.131 (-0.11;0.36)	0.294		0.027 (-0.23;0.18)	0.798		0.143 (-0.01;0.29)	0.064
Smoking n (%)	0.012 (-1.65;1.97)	0.861		0.019 (-1.53;2.03)	0.782		0.015 (-1.59;1.99)	0.825
Alcohol n (%)	-0.054 (-2.20;0.96)	0.442		-0.007(-2.33;0.79)	0.330		-0.051 (-2.14;0.98)	0.464

*Abbreviations*: *WC* Waist circumference, *MUAC* Mid upper arm circumference, *NC* Neck circumference, *MAP* Mean arterial pressure, *BMI* Body mass index

## Discussion

In routine clinical practice, it is a common practice to measure blood pressure in one arm. However, this approach overlooks the variations in blood pressure that usually occur between the arms. These variations in blood pressure between arms are crucial to take note of as they have been identified as a precursor of vascular diseases, in addition to aiding in the detection of vascular diseases, cautious observation of IAD is essential for ensuring accurate diagnosis [3, 9]. The present study investigated the proportion of IAD and its association with body circumferences in the general population of the WSU community. The proportion of IAD was found to be 14.78% among the WSU community. Furthermore, a statistically significant positive association between IAD and body circumference was observed.

The proportion of IAD in this study was higher than a previous report by Sebati et al. [24] which reported a prevalence of 1.92% in the Limpopo province of South Africa. However, it should be noted that the study exclusively included individuals aged 18 to 29, while our research incorporated individuals aged 18 and above, without imposing any age restriction. However, this study is similar to a previous report by Grossman et al. [25] which presented a 12.1% prevalence in the general population of Israel. Furthermore, these results are in accordance with a meta-analysis that reported a prevalence of 14% [26]. However, in a study conducted by Seethalakshmi et al. [27] the prevalence of IAD was much higher, 46% of the participants had IAD in systolic BP of > 10 mmHg. This high prevalence may be due to the fact that more than half of the percentage presented a family history of hypertension. Nevertheless, our findings suggest that IAD is also common in the WSU community.

In this study, an independent positive association between IAD in SBP with MUAC and NC only, was observed, after adjusting for covariates. These findings are in accordance with a study carried out in Finland on a sample of 484 Finnish adults aged between 25 to 74 years that reported a positive association of IAD with MUAC [10]. Furthermore, a study by Muñoz Torrez et al. [20] reported a significant positive association between IAD and adiposity measures, NC, MUAC, WC, and thigh circumference where NC showed the strongest association with IAD of both systolic and diastolic BP. Our study also reported similar findings as NC and MUAC showed a strong association with IAD of SBP after being adjusted for other covariates including the other body circumferences. However, there was no association between WC and IAD, this may indicate that peripheral adiposity, measured by MUAC and NC is more strongly correlated to IAD than central adiposity, measured by WC.

There has not yet been a direct mechanism established that associates IAD with body circumferences. However, it is widely known that body circumferences, particularly MUAC and NC, are measures of upper body subcutaneous fat [28, 29]. Empirical evidence indicated by studies has shown that subcutaneous adipose tissue in the upper body is the primary origin of circulating free fatty acids, and it is a strong predictor of insulin resistance [9, 30, 31]. Additionally, the aforementioned factors are responsible for the onset of atherosclerosis, a common cause of vascular diseases, in which it has been proven that a high IAD is a precursor of these diseases [32]. Taken in combination, these findings imply that greater MUAC and NC may serve as significant indicators of high IAD due to their ability to assess upper body subcutaneous adipose tissue. This further highlights the importance of exploring upper body subcutaneous adipose tissue as a significant pathogenic adipose tissue store.

There were certain limitations to this study, as the current study was a cross-sectional study, no causal inference can be drawn. Secondly, although we have adjusted for multiple confounders, family medical history, food consumption, lipid profile, and physical activity which we did not include might influence these findings. However, we suggest that future studies incorporate these variables into linear regression models in order to confirm our findings among the University communities. Additionally, this study used anthropometric indices to measure upper body subcutaneous fat, therefore we suggest that future studies utilize the direct equipment to measure upper body subcutaneous fat. Lastly, this study was limited to the WSU community, the generalisability of our findings to other demographic and ethnic communities should be approached with caution.

## Conclusion

This study established the proportion of IAD among the WSU community. Furthermore, this study provided evidence of a positive association between body circumferences and IAD. Our findings emphasize the importance of measuring blood pressure on both arms in routine clinical practice, as differences often occur between the arms. Furthermore, our findings suggest that as simple and non-invasive parameters, body circumferences may be utilized as important predictors of high IAD thus aiding in prompt detection and prevention of vascular diseases.

### Supplementary Information

**Supplementary Materials 1. **

## Data Availability

The data that support the findings of this study are accessible from the corresponding author, however access to these data is restricted, because they were used under authorization for the current study and hence are not publicly available.

## Abbreviations

BP: Blood pressure
BMI: Body mass index
CVDs: Cardiovascular diseases
DBP: Diastolic blood pressure
IAD: Interarm blood pressure difference
IADBPD: Interarm diastolic blood pressure difference
IASBPD: Interarm systolic blood pressure difference
MAP: Mean arterial pressure
MUAC: Mid-upper arm circumference
NC: Neck circumference
SBP: Systolic blood pressure
WC: Waist circumference
WSU: Walter Sisulu University
