# Supplementary material for: Association of interarm blood pressure difference with selected body circumferences among Walter Sisulu University community
Source: BMC Public Health. 2024 Feb 29;24:644. doi: 10.1186/s12889-024-18117-5 (PMC10902929; doi:10.1186/s12889-024-18117-5)
Supplement: Supplementary file 1 — Supplementary Materials 1. [file 12889_2024_18117_MOESM1_ESM.docx]

**Association of interarm blood pressure difference with selected body circumferences among Walter Sisulu University community**

**Supplementary Material**

**Table S1**: Distribution of IAD among students and staff members of the WSU community.

|  | SBP (L-R) | | | | DBP (L-R) | | |
| --- | --- | --- | --- | --- | --- | --- | --- |
|  | <10 mmHg | ≥ 10 mmHg | P value |  | <10 mmHg | ≥ 10 mmHg | P value |
| Males n (%) | 100 (85. 5) | 17 (14. 5) | 0.530 |  | 110 (94.0) | 7 (6.0) | 0.181 |
| Females n (%) | 96 (85.0) | 17 (15) |  |  | 110 (97.3) | 3 (2.7) |  |
| Students **(**18-27) n(%) | 163 (88.1) | 22 (11.9) | 0.018 |  | 179 (96.8) | 6 (3.2) | 0.109 |
| Staff (18-64) n (%) | 33(73.3) | 12(26.7) |  |  | 41 (91.1) | 4 (8.9) |  |

Abbreviations: SBP (L-R) interarm difference in systolic blood pressure, DBP (L-R) interarm difference in diastolic blood pressure

**Table S2** Independent association between interarm diastolic blood pressure as dependent variable and WC, MUAC and WC as independent variables.

|  | Interarm diastolic blood pressure | | | | | | | |
| --- | --- | --- | --- | --- | --- | --- | --- | --- |
|  | Adjusted R^2^ =0.037 | | Adjusted R^2^ =0.036 | | | Adjusted R^2^ =0.033 | | |
|  | β (95% CI) | P-value |  | β (95% CI) | P-value |  | β (95% CI) | P-value |
| WC (cm) | 0.211 (-0.02;0.12) | 0.155 | MUAC (cm) | 0.137 (-0.06;0.26) | 0.202 | NC (cm) | 0.078 (-0.04;0.14) | 0.295 |
| MAP (mmHg) | 0.073 (-0.02;0.06) | 0.345 |  | 0.079 (-0.02;0.07) | 0.302 |  | 0.068 (-0.03;0.06) | 0.395 |
| Age (years) | 0.107 (-0.02;0.12) | 0.197 |  | 0.132 (-0.01;0.11) | 0.094 |  | 0.047 (-0.01;0.12) | 0.070 |
| Gender (male) | -0.103 (-1.64;0.26) | 0.154 |  | -0.115 (-1.69;0.19) | 0.115 |  | 0.079 (-1.66;0.27) | 0.157 |
| BMI (kg/m^2^) | -0.240 (-0.29;0.01) | 0.063 |  | -0.182 (-0.24;0.01) | 0.080 |  | 0.138 (-0.15;0.03) | 0.172 |
| Smoking n (%) | 0.002 (-1.11;1.07) | 0.975 |  | 0.001 (-1.09;1.10) | 0.993 |  | 0.201 (-1.10;1.08) | 0.986 |
| Alcohol n (%) | -0.028 (-1.14;0.77) | 0.697 |  | -0.029 (-1.16;0.76) | 0.684 |  | -0.581 (-1.10;0.81) | 0.770 |

Abbreviations: WC waist circumference; MUAC, mid-upper arm circumference; NC, neck circumference; MAP, mean arterial pressure; BMI, body mass index

**Table S3** Independent association of IAD as dependent variable with body circumferences as independent variables

|  | Interarm systolic blood pressure | | Interarm diastolic blood pressure | | |
| --- | --- | --- | --- | --- | --- |
|  | Adjusted R^2^ =0.130 | | Adjusted R^2^ =0.036 | | |
|  | β (95% CI) | P-value | β (95% CI) | | P-value |
| WC (cm) | 0.035 (-0.14;0.11) | 0.794 |  | 0.167 (-0.03; 0.12) | 0.243 |
| MUAC (cm) | 0.241 (0.03; 0.60) | 0.031 |  | 0.071 (-0.12;0.23) | 0.547 |
| NC (cm) | 0.112 (-0.04;0.28) | 0.129 |  | 0.009 (-0.05;0.14) | 0.374 |
| MAP (mmHg) | 0.143 (0.00; 0.14) | 0.061 |  | 0.950 (-0.03; 0.06) | 0.533 |
| Age (years) | 0.053 (-0.07;0.14) | 0.502 |  | 0.099 (-0.03;0.10) | 0.234 |
| Gender (male) | 0.013 (-1.45;1.75) | 0.853 |  | -0.081 (-1.53;0.44) | 0.276 |
| BMI (kg/m^2^) | 0.000 (-0.25;0.25) | 0.998 |  | -0.286 (-0.31; -0.01) | 0.039 |
| Smoking n (%) | 0.020 (-1.51;2.05) | 0.765 |  | 0.001 (-1.09;1.10) | 0.989 |
| Alcohol n (%) | -0.063 (-2.29;0.83) | 0.359 |  | -0.031 (-1.16;0.75) | 0.673 |

Abbreviations: WC waist circumference; MUAC, mid upper arm circumference; NC, neck circumference; MAP, mean arterial pressure; BMI, body mass index
